# Kidney Function-Specific Performance of High-Sensitivity Troponin T and I Using 0/1 h and 0/3 h Protocols in Suspected Non-ST-Segment Elevation Acute Coronary Syndrome

**DOI:** 10.3390/biomedicines14061360

**Published:** 2026-06-17

**Authors:** Krongkarn Sutham, Boriboon Chenthanakij, Aumarin Kumpool, Theerapon Tangsuwanaruk, Arintaya Phrommintikul, Borwon Wittayachamnankul, Rudklao Sairai, Wachira Wongtanasarasin

**Affiliations:** 1 Department of Emergency Medicine, Faculty of Medicine, Chiang Mai University, Chiang Mai 50200, Thailand; krongkarn.s@cmu.ac.th (K.S.); boriboon.c@cmu.ac.th (B.C.); aumarindear@gmail.com (A.K.); theerapon.t@cmu.ac.th (T.T.); borwon.witt@cmu.ac.th (B.W.); rudklao.s@cmu.ac.th (R.S.); 2 Acute Care and Emergency Medicine (ACE) Research Cluster, Faculty of Medicine, Chiang Mai University, Chiang Mai 50200, Thailand; 3 Division of Cardiology, Department of Internal Medicine, Faculty of Medicine, Chiang Mai University, Chiang Mai 50200, Thailand; arintaya.p@cmu.ac.th

**Keywords:** chronic kidney disease, acute coronary syndrome, troponin I, troponin T, emergency service

## Abstract

**Background/Objectives**: Impaired kidney function is associated with persistently elevated cardiac troponin levels, complicating evaluation of suspected non-ST-segment elevation acute coronary syndrome (NSTE-ACS). The comparative performance of high-sensitivity cardiac troponin T (hs-cTnT) and I (hs-cTnI) across sampling intervals in this population remains uncertain. We aimed to identify a kidney function-adapted assay-sampling protocol combination for suspected NSTE-ACS that may support collaborative pathways between nephrologists and acute care clinicians. We therefore assessed kidney function-specific diagnostic and prognostic performance using 0/1 h and 0/3 h protocols. **Methods**: We conducted a prospective observational cohort study of adults presenting with suspected NSTE-ACS at a tertiary emergency department between March 2019 and December 2020. Patients were stratified according to kidney function at presentation using estimated glomerular filtration rate (eGFR). Impaired kidney function was operationally defined as eGFR < 60 mL/min/1.73 m^2^. Serial hs-cTnT and hs-cTnI concentrations were measured at 0, 1, and 3 h and interpreted using assay-specific thresholds and delta criteria. Diagnostic performance for NSTE-ACS and prognostic performance for 30-day major adverse cardiovascular events (MACEs) were evaluated. **Results**: Among 140 patients, 58 (41%) had impaired kidney function. Baseline hs-cTnT and hs-cTnI concentrations were significantly higher in patients with impaired kidney function across all sampling time points. In this group, the 0/3 h protocol demonstrated superior diagnostic performance compared with the 0/1 h protocol for both assays. Using 0/3 h testing, hs-cTnI achieved the highest sensitivity (88.6%; 95% CI, 49.2–95.3), whereas hs-cTnT showed the highest negative predictive value (92.2%; 95% CI, 76.2–94.6). In patients with preserved kidney function, both assays demonstrated high specificity and positive predictive value with the 0/3 h protocol. Prognostic discrimination for 30-day MACEs also improved with a 0/3 h strategy, particularly in patients with impaired kidney function. **Conclusions**: In patients with impaired kidney function and suspected NSTE-ACS, extending troponin testing to 3 h improves diagnostic accuracy and short-term prognostic performance, supporting kidney function-adapted troponin strategies in emergency and nephrology care.

## 1. Introduction

Acute coronary syndrome is a leading cause of death in patients with chronic kidney disease (CKD), and cardiovascular complications frequently determine prognosis across all stages of impaired kidney function [1,2,3]. CKD affects approximately 10–15% of the global adult population and is associated with substantially increased cardiovascular morbidity and mortality [4]. Patients with impaired kidney function commonly present with non-ST-segment elevation acute coronary syndrome (NSTE-ACS), yet diagnosis remains challenging because cardiac troponin concentrations are often chronically elevated even in the absence of acute myocardial ischemia [5,6]. These persistent elevations may reflect structural myocardial remodeling, chronic inflammation, microvascular disease, volume overload, and reduced renal clearance, thereby decreasing the specificity of conventional high-sensitivity troponin thresholds for acute myocardial infarction in this high-risk population [6,7]. Furthermore, the interpretation of elevated troponin levels in patients with impaired kidney function remains clinically challenging. It may contribute to diagnostic uncertainty, unnecessary invasive investigations, prolonged hospitalization, and delayed treatment decisions.

High-sensitivity cardiac troponin (hs-cTn) assays, specifically hs-cTnT and hs-cTnI, are central to current diagnostic strategies for acute myocardial infarction and are recommended by the European Society of Cardiology (ESC) and American Heart Association guidelines for rapid rule-in and rule-out of NSTE-ACS [8,9,10]. Serial measurement protocols—most notably the 0/1 h and 0/3 h algorithms—have been widely validated in general emergency department (ED) populations [8,10]. However, their comparative diagnostic performance in patients with impaired kidney function remains uncertain [11]. In addition, there is ongoing debate over whether hs-cTnT or hs-cTnI provides superior diagnostic and prognostic performance in renal dysfunction, given their distinct biological and analytical characteristics [12]. Previous studies have also suggested that chronically elevated baseline troponin concentrations may reduce specificity and positive predictive value, potentially leading to overdiagnosis and unnecessary interventions in this population [13,14,15].

Despite the widespread clinical implementation of high-sensitivity troponin algorithms, direct head-to-head comparisons of hs-cTnT and hs-cTnI across different serial sampling protocols in patients with impaired kidney function remain limited [16,17]. In particular, evidence comparing the diagnostic and prognostic performance of 0/1 h versus 0/3 h algorithms within the same prospective cohort is scarce, especially in patients with chronically elevated baseline troponin concentrations. Clarifying kidney function-specific assay performance may improve diagnostic accuracy, reduce unnecessary invasive investigations, and support more individualized collaboration between emergency physicians, cardiologists, and nephrologists.

In this prospective cohort study, we directly compared the diagnostic accuracy and short-term prognostic utility of hs-cTnT and hs-cTnI using both 0/1 h and 0/3 h serial sampling protocols in patients with and without impaired kidney function who presented with suspected NSTE-ACS. By measuring both assays in the same individuals and stratifying by kidney function, we sought to minimize between-patient variability and delineate kidney function-specific patterns in sensitivity, specificity, and prediction of major adverse cardiac events [9]. We aimed to identify an assay-and-protocol combination better tailored to patients with impaired kidney function and potentially applicable within collaborative nephrology-and-acute-care pathways.

## 2. Materials and Methods

### 2.1. Study Design and Setting

We conducted a prospective cohort study in the ED of a 1500-bed tertiary referral center in Northern Thailand between March 2019 and December 2020. The ED evaluates approximately 40,000 patients annually and has on-site availability of both hs-cTnT and hs-cTnI assays. The study adhered to the Standards for Reporting Diagnostic Accuracy Studies (STARD) recommendations for diagnostic accuracy research [18]. The study was conducted in accordance with the Declaration of Helsinki and was approved by the Research Ethics Committee 4, Faculty of Medicine, Chiang Mai University (Certificate of Approval No. 058/2019). Written informed consent was obtained from all participants prior to enrollment in the study.

### 2.2. Participants and Kidney Function Classification

We prospectively enrolled consecutive adults aged ≥18 years who presented to the ED with symptoms suggestive of acute coronary syndrome, including chest pain, dyspnea, chest discomfort, epigastric pain, diaphoresis, syncope, or angina-equivalent symptoms, without persistent ST-segment elevation on the initial 12-lead electrocardiogram (ECG). Patients were stratified according to kidney function at presentation using estimated glomerular filtration rate (eGFR). To avoid potential misclassification of CKD based solely on a single creatinine measurement, the terms “impaired kidney function” and “preserved kidney function” were used operationally in this study rather than definitive CKD classification.

- Impaired kidney function group: eGFR < 60 mL/min/1.73 m^2^ at presentation;
- Preserved kidney function group: eGFR ≥ 60 mL/min/1.73 m^2^ at presentation.

Kidney function classification was interpreted in accordance with Kidney Disease: Improving Global Outcomes (KDIGO) 2024 criteria [19]. However, because data on chronicity ≥3 months and albuminuria were not systematically available for all participants, definitive diagnosis and staging of CKD according to KDIGO criteria could not be established in all cases.

#### 2.2.1. Inclusion Criteria

Eligible participants were consecutive adult patients presenting with suspected NSTE-ACS who underwent serial hs-cTnT and hs-cTnI measurements at 0, 1, and 3 h, in accordance with the study protocol.

#### 2.2.2. Exclusion Criteria

Exclusion criteria included:

- ST-segment elevation myocardial infarction (STEMI);
- Pregnancy;
- Cardiac arrest or cardioversion before blood sampling;
- Known skeletal muscle disease;
- Active systemic infection or sepsis;
- Incomplete serial troponin measurements;
- Refusal to participate;
- Non-Asian ethnicity.

### 2.3. Study Protocol and Troponin Measurements

All patients underwent routine diagnostic and therapeutic evaluation according to institutional ED protocols and contemporary ESC recommendations for suspected NSTE-ACS [20,21]. Structured clinical assessment included symptom evaluation, medical history, physical examination, ECG interpretation, and laboratory testing.

Blood samples for hs-cTnT and hs-cTnI were collected at presentation (0 h) and repeated at 1 and 3 h after ED arrival in accordance with the approved study protocol and informed consent procedures. hs-cTnT was measured using the Roche Elecsys^®^ Troponin T high-sensitivity STAT assay (99th percentile upper reference limit [URL]: 14 ng/L), and hs-cTnI was calculated using the Abbott Architect^®^ STAT High Sensitivity Troponin I assay (99th percentile URL: 34.2 ng/L for men, 15.6 ng/L for women, and 26.2 ng/L overall). A significant troponin delta change was defined according to ESC recommendations [20,21]:

- ≥50% relative change for baseline values below the 99th percentile URL;
- ≥20% relative change for baseline values above the 99th percentile URL.

Two serial sampling algorithms were evaluated in both kidney function groups:

- 0/1 h protocol: Baseline and 1 h hs-cTn values with delta-change interpretation;
- 0/3 h protocol: Baseline and 3 h hs-cTn values with delta-change interpretation.

In addition to standard assay-specific 99th-percentile thresholds, exploratory analyses using approximately 5-fold upper-reference-limit cutoffs were performed to improve specificity in patients with chronically elevated baseline troponin concentrations, based on prior ESC-related literature and previous studies involving populations with renal dysfunction.

Further investigations, including transthoracic echocardiography, coronary angiography with or without percutaneous coronary intervention (PCI), computed tomography, chest radiography, and cardiac magnetic resonance imaging, were performed according to clinical indication and physician judgment rather than systematically in all participants.

### 2.4. Diagnostic Definitions and Adjudication

Patients with suspected NSTE-ACS underwent diagnostic evaluation according to institutional ED protocols and contemporary ESC guidelines [20,21]. Final diagnoses were established using integrated clinical adjudication based on presenting symptoms, serial troponin changes, ECG findings, imaging results, coronary angiographic findings when available, procedural data, and follow-up information.

In this study, NSTE-ACS included both non-ST-segment elevation myocardial infarction (NSTEMI) and unstable angina. NSTEMI was defined according to the Fourth Universal Definition of Myocardial Infarction as evidence of acute myocardial injury with dynamic troponin elevation above the 99th percentile upper reference limit in the setting of clinical evidence of myocardial ischemia. Unstable angina was defined as ischemic symptoms suggestive of ACS without diagnostic troponin elevation fulfilling criteria for myocardial infarction.

Non-ACS cardiac causes included cardiac conditions other than acute coronary syndrome that could explain symptoms or troponin elevation, including heart failure, arrhythmia, myocarditis, hypertensive emergency, or structural heart disease. Non-cardiac causes included pulmonary, gastrointestinal, musculoskeletal, infectious, or other systemic conditions without evidence supporting ACS. Major adverse cardiovascular events (MACEs) were defined as a composite of acute myocardial infarction, urgent coronary revascularization (PCI or coronary artery bypass grafting [CABG]), and all-cause mortality.

Coronary angiography and revascularization decisions were performed according to treating cardiologists’ clinical judgment, guideline-directed management, and real-world practice rather than by a predefined study protocol. When discrepancies in diagnosis occurred or documentation was incomplete, an independent study cardiologist reviewed all available clinical, laboratory, imaging, and procedural data to establish a consensus diagnosis.

### 2.5. Study Outcomes

The primary outcome was the diagnostic performance of cardiac troponins (hs-cTnT and hs-cTnI) using both 0/1 and 0/3 protocols for identifying NSTE-ACS in patients with impaired kidney function, including sensitivity, specificity, positive predictive value (PPV), negative predictive value (NPV), likelihood ratios, and overall diagnostic accuracy. The secondary outcome was prognostic performance for 30-day MACEs, defined as the composite of acute myocardial infarction, urgent coronary revascularization, and all-cause mortality.

### 2.6. Statistical Analysis

Sample size estimation was performed using receiver operating characteristic (ROC) curve methodology based on previously reported hs-cTn diagnostic performance. Using an anticipated area under the ROC of 0.93 derived from prior studies [22], accounting for incomplete serial troponin measurements and potential loss to follow-up, the targeted enrollment sample size was 140. Continuous variables were reported as mean ± standard deviation or median with interquartile range, as appropriate, and compared using Student’s *t*-test or Mann–Whitney U test. Categorical variables were presented as counts and percentages and compared using chi-square or Fisher’s exact test. Diagnostic performance metrics, including sensitivity, specificity, PPV, NPV, likelihood ratios, and accuracy, were calculated with 95% confidence intervals. ROC analyses were performed to evaluate prognostic performance for 30-day MACEs. A two-sided *p*-value < 0.05 was considered statistically significant. Analyses were performed using Stata/MP version 14.1 (StataCorp, College Station, TX, USA).

## 3. Results

**Figure 1:**
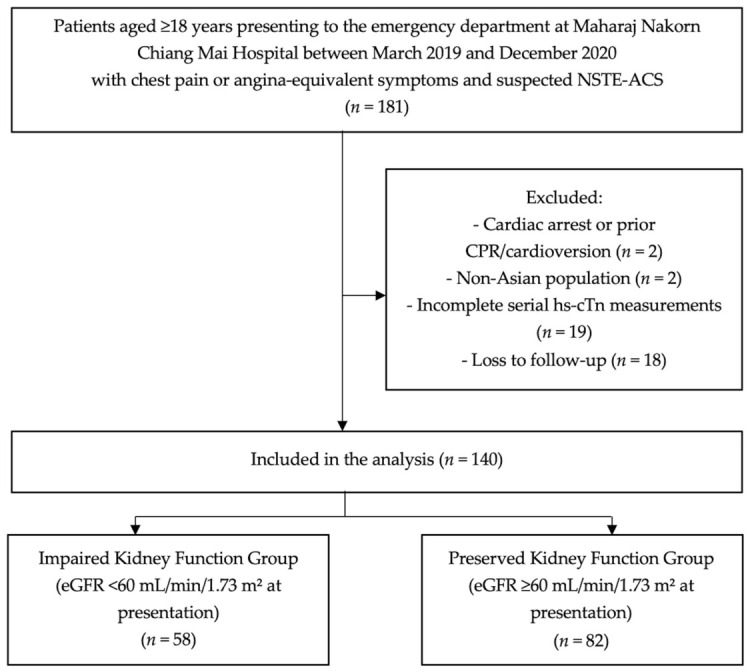
Study flow diagram. Abbreviations: CPR, cardiopulmonary resuscitation; NSTE-ACS, non-ST-segment elevation acute coronary syndrome.

A total of 140 patients presenting with suspected NSTE-ACS were included in the final analysis (Figure 1). Based on kidney function at presentation, 58 patients (41.4%) had impaired kidney function (median eGFR 29.2 mL/min/1.73 m^2^), and 82 patients (58.6%) had preserved kidney function. Patients with impaired kidney function were older and had higher prevalences of hypertension and diabetes compared with those with preserved kidney function. They also more frequently presented with higher Killip classifications, suggesting greater hemodynamic severity at presentation. Patients categorized as ‘unknown’ had insufficient diagnostic information or incomplete follow-up to allow definitive adjudication of ACS versus non-ACS etiologies (Table 1).

Median baseline hs-cTnT and hs-cTnI levels were significantly higher in patients with impaired kidney function across all sampling time points (*p* < 0.01). NSTE-ACS was diagnosed in 17.2% of patients with impaired kidney function compared with 11.0% of those with preserved kidney function (*p* = 0.02). In patients with impaired kidney function, the 0/3 h protocol demonstrated superior diagnostic performance compared with the 0/1 h algorithm for both assays. Using the 0/3 h protocol, hs-cTnI achieved the highest sensitivity (88.6%, 95% CI 49.2–95.3%), whereas hs-cTnT demonstrated the highest NPV (92.2%, 95% CI 76.2–94.6%). However, specificity (67.4–74.4%) and PPV (44.0–51.7%) remained modest, likely reflecting chronically elevated baseline troponin concentrations in this population (Table 2).

In patients with preserved kidney function, both assays demonstrated high specificity (>96%) and PPV (>80%) using the 0/3 h protocol. hs-cTnI achieved 100% specificity and PPV, with a sensitivity of 96.3%. Positive likelihood ratios for hs-cTnI exceeded 20, indicating strong rule-in performance, while NPVs remained above 90% for both assays (Table 3). All-cause mortality occurred exclusively among patients with impaired kidney function, with rates of 5.2% at 30 days and 10.3% at 1 year. However, there were no statistically significant differences in 30-day and 1-year MACEs between the impaired- and preserved-kidney function groups (Table 4).

**Figure 2:**
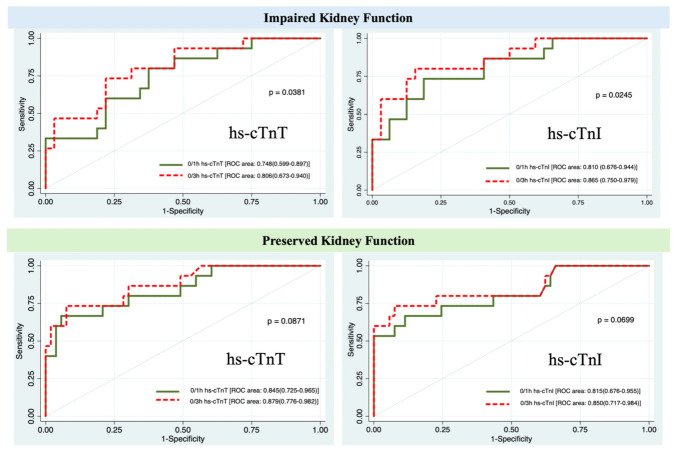
Receiver operating characteristic curves of 0/1 h and 0/3 h troponin measurement protocols for 30-day major adverse cardiovascular events. Abbreviations: hs-cTnI, high-sensitivity cardiac troponin I; hs-cTnT, high-sensitivity cardiac troponin T; ROC, receiver operating characteristic.

Figure 2 illustrates the prognostic performance of hs-cTnT and hs-cTnI for predicting 30-day MACEs according to kidney function status. Both assays demonstrated excellent prognostic discrimination with the 0/3 h protocol, regardless of kidney function, with area under the ROC (AUC) values exceeding 0.8. In patients with impaired kidney function, extending the sampling interval from 0/1 h to 0/3 h significantly improved short-term prognostic performance for both hs-cTnT (*p* = 0.04) and hs-cTnI (*p* = 0.02). In contrast, among patients with preserved kidney function, the incremental prognostic benefit of the 0/3 h protocol was smaller and did not reach statistical significance for either hs-cTnT (*p* = 0.09) or hs-cTnI (*p* = 0.07).

## 4. Discussion

In this prospective cohort of patients presenting with suspected NSTE-ACS, kidney function substantially influenced both the diagnostic and prognostic performance of high-sensitivity troponin assays. Patients with impaired kidney function demonstrated markedly elevated baseline hs-cTnT and hs-cTnI concentrations, resulting in reduced specificity and PPV when rapid 0/1 h algorithms were applied. Extending serial sampling to 3 h appeared to improve diagnostic discrimination and short-term prognostic performance, particularly for hs-cTnI. These findings support the need for a kidney function-adapted interpretation of high-sensitivity troponin rather than applying a single universal strategy across heterogeneous renal populations.

Consistent with previous studies [15], baseline troponin concentrations were substantially higher among patients with impaired kidney function, reflecting the challenge of distinguishing chronic myocardial injury from acute ischemic events in this population. Chronic troponin elevation in renal dysfunction is likely multifactorial and may reflect structural myocardial remodeling, chronic inflammation, microvascular ischemia, and reduced renal clearance [13,14,15]. In our cohort, this baseline shift reduced specificity and PPV for both hs-cTnT and hs-cTnI, although NPVs remained high. These findings are clinically relevant because false-positive troponin interpretation may contribute to unnecessary invasive procedures and prolonged hospitalization.

Our findings also contribute to the ongoing debate regarding the comparative performance of hs-cTnT and hs-cTnI in patients with impaired kidney function. Prior studies have reported conflicting results, with some demonstrating superior specificity of hs-cTnI and others reporting greater sensitivity with hs-cTnT [9,23]. In our cohort, hs-cTnI demonstrated modestly improved specificity and overall diagnostic balance, particularly when combined with the 0/3 h protocol, whereas hs-cTnT retained slightly higher sensitivity in some analyses. These differences may reflect underlying biological and analytical differences between the assays [24,25,26]. hs-cTnT appears more strongly influenced by structural heart disease and chronic myocardial remodeling. In contrast, hs-cTnI may demonstrate more dynamic changes during acute injury, potentially improving discrimination between chronic and acute troponin elevation [9,27].

Importantly, the 0/3 h algorithm consistently outperformed the 0/1 h protocol in patients with impaired kidney function across both diagnostic and prognostic analyses. Extending the sampling improved discrimination and reduced false-positive classification attributable to chronically elevated baseline troponin concentrations [28,29]. Although rapid rule-out algorithms are attractive in emergency department workflows, abbreviated protocols may be less reliable in patients with impaired kidney function because elevated baseline troponin levels can obscure acute dynamic changes.

An important strength of this study is the direct head-to-head comparison of hs-cTnT and hs-cTnI using both 0/1 h and 0/3 h protocols within the same prospective cohort. By simultaneously measuring both assays in the same patients and stratifying analyses by kidney function, we minimized between-patient variability and better characterized kidney function-specific patterns of assay performance. These findings may help support kidney function-adapted troponin strategies in emergency and nephrology practice.

### 4.1. Clinical Implications

From a clinical perspective, our findings suggest that a more conservative 0/3 h serial testing strategy may be preferable in patients with impaired kidney function presenting with suspected NSTE-ACS, particularly when baseline troponin concentrations are elevated and the pretest probability of ACS is intermediate. In this setting, hs-cTnI combined with a 0/3 h protocol demonstrated a favorable balance between sensitivity and specificity while maintaining strong prognostic discrimination for short-term MACEs. Incorporating kidney function-adapted troponin interpretation strategies into institutional chest pain pathways may reduce diagnostic misclassification and improve interdisciplinary decision-making among emergency physicians, cardiologists, and nephrologists [30].

### 4.2. Limitations and Future Directions

Several limitations should be considered. First, this was a single-center study conducted in a tertiary referral ED, and the study population consisted exclusively of Asian patients; therefore, the findings may not be fully generalizable to other healthcare settings or ethnic populations. Second, definitive CKD diagnosis and staging according to KDIGO criteria could not be established for all participants because data on chronicity and albuminuria were not systematically available. Accordingly, kidney function groups should be interpreted operationally rather than as definitive CKD classifications.

Third, the relatively modest sample size and limited number of NSTE-ACS and MACEs may have reduced statistical precision and limited subgroup analyses according to kidney function severity. Adjustment for age, cardiovascular comorbidities, dialysis status, and structural heart disease was also constrained by data availability and sample size. In addition, detailed echocardiographic parameters were not systematically available for all participants, and conditions such as heart failure or left ventricular hypertrophy may have influenced baseline troponin concentrations. Therefore, the findings should be interpreted as exploratory and hypothesis-generating until validated in larger multicenter cohorts.

Fourth, coronary angiography was not systematically performed in all patients because clinical indications and real-world physician decision-making guided investigations. Although this reflects routine practice, some degree of diagnostic misclassification remains possible. Adjudicators were also not blinded to troponin results, which may have introduced incorporation bias.

Finally, troponin measurements were performed using specific Roche^®^ and Abbott^®^ assay platforms; diagnostic performance may differ from that of other commercially available assays due to analytical variability. Future multicenter studies with larger and more diverse populations are needed to validate kidney function-adapted troponin algorithms and refine assay-specific cutoff strategies in patients with impaired kidney function.

## 5. Conclusions

In patients with impaired kidney function presenting with suspected NSTE-ACS, kidney function substantially influenced the diagnostic and prognostic performance of high-sensitivity troponin assays. A 0/3 h serial testing protocol, particularly when using hs-cTnI, demonstrated a more favorable balance between sensitivity, specificity, and short-term MACE prediction compared with the rapid 0/1 h protocol. These findings support a kidney function-adapted troponin interpretation strategy rather than a single universal high-sensitivity troponin algorithm across the spectrum of renal function. Given the exploratory nature of this study, larger multicenter investigations are needed to validate these findings and refine assay-specific strategies in patients with impaired kidney function.

## Figures and Tables

**Table 1:** Baseline characteristics of patients with suspected non-ST-elevation acute coronary syndrome.

Characteristics	All Patients (*n* = 140)	Impaired Kidney Function (*n* = 58)	Preserved Kidney Function (*n* = 82)	*p*-Value *
Age, years—mean (SD)	65.9 (15.0)	73.8 (11.3)	60.3 (14.8)	**<0.001**
Sex—female, n (%)	65 (46)	23 (40)	42 (51)	0.18
Comorbidities, n (%)				
Hypertension	80 (57)	40 (69)	40 (49)	**0.02**
Diabetes	38 (27)	24 (41)	14 (17)	**<0.001**
Dyslipidemia	53 (38)	25 (43)	28 (34)	0.28
Coronary arterial disease	55 (39)	28 (48)	27 (33)	0.07
Initial vital signs, mean (SD)				
SBP—mmHg	141.7 (29.8)	141.8 (30.8)	141.6 (29.3)	0.98
DBP—mmHg	79.8 (19.1)	76.1 (21.5)	82.4 (16.8)	0.06
Heart rate—beats per minute	86.1 (20.2)	83.4 (21.6)	88.0 (19.0)	0.19
Respiratory rate—per minute	22.6 (8.5)	24.5 (8.5)	21.2 (8.2)	**0.02**
Oxygen saturation—%	95.5 (9.0)	93.1 (13.4)	97.2 (2.4)	**<0.001**
Body temperature—degreeS Celsius	36.4 (2.8)	36.2 (4.4)	36.6 (0.4)	0.40
Chest pain onset—hours, median (IQR)	3 (2–12)	3 (1–21)	3.5 (2–12)	0.74
Killip’s classification, n (%)				**<0.001**
I	105 (85)	35 (60)	70 (85)	
II	28 (20)	16 (28)	12 (15)	
III	3 (2)	3 (5)	0 (0)	
IV	0 (0)	0 (0)	0 (0)	
Initial ECG, n (%)				0.18
Normal	56 (40)	20 (34)	36 (44)	
Significant ST-T abnormalities	24 (17)	7 (12)	17 (21)	
Non-specific ST-T abnormalities	38 (27)	20 (34)	18 (22)	
Same as previous ECG (no changes)	22 (14)	11 (19)	11 (13)	
Initial laboratory results, mean (SD)				
WBC—10^9^/L	8.1 (3.7)	8.4 (4.2)	7.9 (3.3)	0.46
eGFR—mL/min·1.73 m^2^	62.9 (33.2)	29.2 (16.5)	86.8 (17.5)	**<0.001**
Troponin levels (ng/L), median (IQR)				
hs-cTnI at 0 h (baseline)	14.8 (5.1–48.9)	27.5 (10.7–102.7)	7.6 (3.3–21.9)	**<0.001**
hs-cTnI at 1 h	14.4 (5.1–55.7)	27.6 (11.2–130.0)	8.0 (3.5–26.1)	**<0.001**
hs-cTnI at 3 h	16.0 (6.1–80.9)	33.0 (13.4–147.6)	9.2 (4.0–29.4)	**0.04**
hs-cTnT at 0 h (baseline)	20.4 (8.6–41.9)	32.0 (19.9–88.4)	10.1 (6.8–23.7)	**<0.001**
hs-cTnT at 1 h	20.0 (8.8–41.7)	37.8 (18.5–86.1)	10.5 (6.2–22.1)	**0.02**
hs-cTnT at 3 h	20.7 (9.1–44.5)	35.5 (19.3–97.1)	12.0 (7.5–22.8)	**0.03**
Final diagnosis, n (%)				**0.02**
NSTE-ACS	19 (14)	10 (17)	9 (11)	
Unstable angina	11 (8)	4 (7)	7 (9)	
Non-ACS cardiac cause	32 (23)	19 (33)	13 (16)	
Non-cardiac cause	42 (30)	9 (16)	33 (40)	
Unknown	36 (26)	16 (28)	20 (24)	

Percentages have been rounded and may not equal 100. * Statistically significant *p*-values were expressed in bold. Abbreviations: ACS, acute coronary syndrome; ECG, electrocardiogram; eGFR, estimated glomerular filtration rate; WBC, white blood cell; IQR, interquartile range; SD, standard deviation; ECG, electrocardiography; hs-cTnI, high-sensitivity cardiac troponin I; hs-cTnT, high-sensitivity cardiac troponin T; NSTE-ACS, non-ST-segment elevation acute coronary syndrome.

**Table 2:** Diagnostic performance of 0/1 h and 0/3 h troponin measurement protocols for diagnosis of NSTE-ACS in patients with *impaired kidney function*.

	Cutoff (ng/L)	Sensitivity (95% CI)	Specificity (95% CI)	PPV (95% CI)	NPV (95% CI)	LR+ (95% CI)	LR− (95% CI)	Accuracy (95% CI)
**0/1 h protocol**								
hs-cTnT	A	78.6 (35.1–87.2)	67.4 (52.4–81.4)	44.0 (26.4–53.5)	90.6 (74.3–92.6)	2.4 (1.1–3.6)	0.3 (0.3–1.1)	70.2 (53.7–79.0)
hs-cTnI	B	71.4 (41.9–91.6)	72.2 (45.5–75.6)	47.6 (26.3–49.2)	88.9 (74.1–94.1)	2.8 (1.1–3.0)	0.4 (0.2–1.1)	73.7 (50.1–76.0)
**0/3 h protocol**								
hs-cTnT	C	81.4 (41.9–91.6)	68.2 (62.4–82.3)	51.7 (29.3–55.2)	92.2 (76.2–94.6)	2.2 (1.3–3.9)	0.4 (0.2–1.0)	69.0 (55.5–80.5)
hs-cTnI	D	88.6 (49.2–95.3)	74.4 (47.8–77.6)	50.7 (29.9–52.6)	90.3 (77.0–96.3)	2.2 (1.3–3.5)	0.3 (0.1–0.9)	76.9 (53.7–79.0)

Abbreviations: CI, confidence interval; hs-cTnI, high-sensitivity cardiac troponin I; hs-cTnT, high-sensitivity cardiac troponin T; LR, likelihood ratio; NPV, negative predictive value; PPV, positive predictive value; NSTE-ACS, non-ST segment elevation acute coronary syndrome. A: 52 or delta change ≥5 ng/L. B: 64 or delta change ≥6 ng/L. C: 70* or delta change ≥50% of baseline value (<14 ng/L) or delta change ≥20% of positive initial level. D: 131* or delta change ≥50% of baseline value (<26.2 ng/L) or delta change ≥20% of positive initial level. * 5-fold of the 99th percentile of the upper reference limit refers to the conventional assay-specific cutoff for the diagnosis of AMI in healthy individuals, as recommended in the 2015 ESC clinical practice guidelines.

**Table 3:** Diagnostic performance of 0/1 h and 0/3 h troponin measurement protocols for diagnosis of NSTE-ACS in patients with *preserved kidney function*.

	Cutoff (ng/L)	Sensitivity (95% CI)	Specificity (95% CI)	PPV (95% CI)	NPV (95% CI)	LR+ (95% CI)	LR− (95% CI)	Accuracy (95% CI)
**0/1 h protocol**								
hs-cTnT	A	56.3 (24.7–75.4)	96.7 (89.5–99.6)	81.8 (48.4–94.5)	90.0 (83.0–92.1)	18.3 (3.9–70.4)	0.5 (0.3–0.8)	88.9 (78.7–94.0)
hs-cTnI	B	62.5 (35.4–84.8)	98.5 (91.8–100)	90.9 (58.0–98.6)	91.6 (85.2–95.3)	41.3 (5.7–299.3)	0.4 (0.2–0.7)	91.5 (83.2–96.5)
**0/3 h protocol**								
hs-cTnT	C	87.5 (85.4–94.8)	96.7 (90.0–99.7)	83.3 (54.8–95.4)	91.4 (85.0–95.3)	20.6 (5.0–85.0)	0.4 (0.2–0.7)	90.2 (81.7–95.7)
hs-cTnI	D	96.3 (89.9–98.3)	100 (94.6–100)	100 (100–100)	90.4 (84.4–94.3)	N/A	0.4 (0.3–0.8)	93.6 (85.2–97.9)

Abbreviations: CI, confidence interval; hs-cTnI, high-sensitivity cardiac troponin I; hs-cTnT, high-sensitivity cardiac troponin T; LR, likelihood ratio; NPV, negative predictive value; PPV, positive predictive value; NSTE-ACS, non-ST segment elevation acute coronary syndrome. A: 52 or delta change ≥5 ng/L. B: 64 or delta change ≥6 ng/L. C: 70* or delta change ≥50% of baseline value (<14 ng/L) or delta change ≥20% of positive initial level. D: 131* or delta change ≥50% of baseline value (<26.2 ng/L) or delta change ≥20% of positive initial level. * 5-fold of the 99th percentile of the upper reference limit refers to the conventional assay-specific cutoff for the diagnosis of AMI in healthy individuals, as recommended in the 2015 ESC clinical practice guidelines.

**Table 4:** The 30-day and 1-year outcomes of the cohort.

Outcomes	All Patients (*n* = 140)	Impaired Kidney Function (*n* = 58)	Preserved Kidney Function (*n* = 82)	*p*-Value
30-day outcome, n (%)				0.36
AMI	26 (19)	16 (28)	10 (16)	
Revascularization (PCI +/− CABG)	5 (4)	3 (5)	2 (2)	
Dead (all-cause)	3 (2)	3 (5)	0 (0)	
1-year outcome, n (%)				0.80
AMI	33 (24)	18 (31)	15 (18)	
Revascularization (PCI +/− CABG)	10 (7)	5 (9)	5 (6)	
Dead (all-cause)	6 (4)	6 (10)	0 (0)	

Percentages have been rounded and may not equal 100. Abbreviations: AMI, acute myocardial infarction; CABG, coronary artery bypass grafting; PCI, percutaneous coronary intervention.

## Data Availability

The data presented in this study are available on request from the corresponding author.

## Abbreviations

The following abbreviations are used in this manuscript:

CKD	chronic kidney disease
NSTE-ACS	non-ST-segment elevation acute coronary syndrome
Hs-cTnT	high-sensitivity cardiac troponin T
Hs-cTnI	high-sensitivity cardiac troponin I
MACE	major adverse cardiovascular events
ESC	European Society of Cardiology
ED	emergency department
ECG	electrocardiogram
KDIGO	Kidney Disease: Improving Global Outcomes
eGFR	estimated glomerular filtration rate
PPV	positive predictive value
NPV	negative predictive value
AMI	acute myocardial infarction
